# An integrative multi-dimensional genetic and epigenetic strategy to identify aberrant genes and pathways in cancer

**DOI:** 10.1186/1752-0509-4-67

**Published:** 2010-05-17

**Authors:** Raj Chari, Bradley P Coe, Emily A Vucic, William W Lockwood, Wan L Lam

**Affiliations:** 1 Department of Integrative Oncology, BC Cancer Research Centre, Vancouver, BC, Canada

## Abstract

**Background:**

Genomics has substantially changed our approach to cancer research. Gene expression profiling, for example, has been utilized to delineate subtypes of cancer, and facilitated derivation of predictive and prognostic signatures. The emergence of technologies for the high resolution and genome-wide description of genetic and epigenetic features has enabled the identification of a multitude of causal DNA events in tumors. This has afforded the potential for large scale integration of genome and transcriptome data generated from a variety of technology platforms to acquire a better understanding of cancer.

**Results:**

Here we show how multi-dimensional genomics data analysis would enable the deciphering of mechanisms that disrupt regulatory/signaling cascades and downstream effects. Since not all gene expression changes observed in a tumor are causal to cancer development, we demonstrate an approach based on multiple concerted disruption (MCD) analysis of genes that facilitates the rational deduction of aberrant genes and pathways, which otherwise would be overlooked in single genomic dimension investigations.

**Conclusions:**

Notably, this is the first comprehensive study of breast cancer cells by parallel integrative genome wide analyses of DNA copy number, LOH, and DNA methylation status to interpret changes in gene expression pattern. Our findings demonstrate the power of a multi-dimensional approach to elucidate events which would escape conventional single dimensional analysis and as such, reduce the cohort sample size for cancer gene discovery.

## Background

Genomic analyses have substantially improved our knowledge of cancer. Gene expression profiling, for example, is utilized to delineate subtypes of breast cancer, and has facilitated the derivation of predictive and prognostic signatures [1-5]. However, not all of the gene expression changes observed are causal to cancer development, and global gene expression analysis alone cannot distinguish between causal and reactive changes. Corresponding alteration at the DNA level is regarded as evidence of causality; for example, gene deletion or gene silencing by methylation. Hence, examining genetic and epigenetic events in conjunction with the changes in gene expression pattern should improve the identification of causal changes that lead to disease phenotype.

Analysis of gene copy number alone has correlated breast cancer genome features with poor prognosis based on the degree of genomic instability observed [6]. In terms of gene discovery, specific genomic regions containing important loci have been shown to be frequently gained or lost [7-11]. Integrative analyses of gene dosage and gene expression in breast cancer have revealed specific genes which are deregulated at the gene expression level as a result of changes in DNA copy number. From a global perspective, studies have shown a broad range in concordance between DNA amplification and overexpression of genes. This variability is attributable to the sensitivity of the methods used in detecting gene copy number and gene expression changes as well as the number of genes examined [12-15]. Conversely, when examining gene overexpression, it was found that only ~10% of the overexpression could be attributable to gene amplification [14]. It is certain that altered gene expression can not only be attributed to disruption of regulatory/signaling cascades and downstream effects, but also to a multitude of causal genetic and epigenetic aberrations.

We reason that by examining multiple genomic dimensions simultaneously, with a dimension representing a genome wide assay measuring DNA level alterations such as gene copy number or DNA methylation, we are likely to achieve the following: (i) explain a greater fraction of the observed gene expression deregulation as compared with explaining expression deregulation using only a single dimension, (ii) improve the discovery of critical oncogenes and tumor suppressor genes (TSGs) by focusing on those genes altered simultaneously at multiple genomic dimensions, and (iii) begin to understand the complex mechanisms of dysregulation of oncogenic pathways. In this study, we demonstrate the power of an integrative genomics approach by performing multi-dimensional analyses (MDA) of the genome, epigenome, and transcriptome of breast cancer cell lines. We illustrate and demonstrate the need for integrative analysis of multiple genomic dimensions by showing the co-operative contribution of DNA mechanisms to explaining differential gene expression. Using a strategy to identify genes exhibiting congruent alteration in copy number, DNA methylation, and allelic (or loss of heterozygosity, LOH) status, which we term multiple concerted disruption (MCD) analysis, we find genes representing key nodes in pathways as well as genes which exhibit prognostic significance. In examining the neuregulin pathway, we observe the variability among samples in the mechanism of dysregulation of this commonly altered breast cancer pathway, highlighting the importance of multi-dimensional analysis of a given pathway in individual tumor samples -- in addition to the conventional approach of identifying loci simply based on frequency of disruption in a cohort. Finally, examining the subset of triple negative breast cancer cell (TNBC) lines, we show that a downstream target of *FGFR2*, a recently implicated oncogene in TNBC, *COL1A1 *is frequently affected by MCD even though in *FGFR2 *itself is rarely affected. Notably, this is the first such in-depth genomic, epigenomic, and transcriptomic analyses of breast cancer.

## Methods

### Data generation and acquisition

Commonly used breast cancer (HCC38, HCC1008, HCC1143, HCC1395, HCC1599, HCC1937, HCC2218, BT474, MCF-7) and non-cancer (MCF10A) cell lines were selected for analyses (Additional File 1). Copy number profiles were obtained from the *SIGMA *database [11,16]. These profiles were generated using a whole genome tiling path microarray CGH platform [17,18]. Expression profiles for BT474 and MCF-7 were obtained from the NCI Cancer Biomedical Informatics Grid (caBIG, https://cabig.nci.nih.gov), MCF10A profile from GEO (GSM254525), and the rest were generated using Affymetrix U133 Plus 2.0 platform at the McGill University and Genome Quebec Innovation Centre. Affymetrix 500 K SNP array data were obtained from caBIG. DNA methylation profiles were generated using the Illumina Infinium methylation platform at the Genomics Lab, Wellcome Trust Centre for Human Genetics. A summary of the sources of all the data used is provided in Additional File 2. Gene expression and methylation data generated were deposited in NCBI GEO (GSE17768 and GSE17769).

### Data processing and normalization

Array CGH data were normalized using a stepwise normalization framework [19]. In addition, data were filtered based on a stringent standard deviation cut-off of 0.075 between replicate spots, with those exceeding this cut-off excluded from further analysis. To identify regions of gain and loss, smoothing and segmentation analysis was performed using *aCGH-Smooth *[20] as previously described [21]. Copy number status for clones which were filtered from above were inferred using neighboring clones within a 1 Mb window.

Affymetrix SNP array data were normalized and genotyped using the "oligo" package in R, specifically using the *crlmm *algorithm for genotyping [22]. Genotype calls whose confidences were less than 0.95 were termed "No Call" (NC). Subsequently, genotype profiles were analyzed using *dChip *[23] and LOH was determined using a panel of 60 normal genotypes from the HapMap dataset [24] as provided by *dChip*, as matching blood lymphoblast profiles were not available. LOH ("L"), Retention ("R"), and No Call ("N") status was determined for every marker in each sample. Analysis parameters used were as specified in the *dChip *manual.

Raw gene expression profiles from all ten cell lines were RMA normalized using the "affy" package in *Bioconductor *[25,26](Additional File 3). Gene expression data were further filtered using the Affymetrix MAS 5.0 Call values ("P","M", and "A"). Since the comparison of differential expression was one cancer line to one normal, both call values could not be "Absent" in order to be retained for analysis.

Methylation data were normalized and processed using *Illumina BeadStudio *software (http://www.illumina.com/software/genomestudio_software.ilmn, Illumina, Inc., San Diego, CA, USA). Beta-values and confidence p-values were retained for further analysis. Beta-values with associated confidence p-values > 0.05 were excluded. Data from all genomic dimensions were mapped to the hg18 (March 2006) genome assembly.

### Strategy for integrative analysis

Copy number and LOH profiles were mapped to genes using the mapping of the Affymetrix U133 Plus 2.0 platform as well as the UCSC Genome Browser [27]. Methylation data were linked to the other three types of data using either the RefSeq gene symbol as specified by the Illumina mapping file (Illumina), or the RefSeq accession number. Differential expression was determined by subtracting the expression value in the non-malignant line MCF10A from the value in each cancer line. Since the obtained gene expression values after RMA normalization were represented in log_2 _space, a gene was considered differentially expressed if the difference between the cancer line and MCF10A was greater than 1, which corresponded to a two-fold expression difference. DNA methylation status was determined by subtracting beta-values, with hypermethylation defined as a positive difference between tumor and normal (≥ 0.25) and hypomethylation defined as a negative difference between tumor and normal (≤ -0.25). Briefly, a beta value for a given CpG site ranges from 0 to 1 and represents the ratio of the methylated signal over the total signal (methylated plus unmethylated signal). These thresholds are comparable to those used in previous studies using an earlier Illumina methylation platform [28]. Using this mapping strategy, 12,910 unique genes were mapped across platforms corresponding to 24,708 of the ~27,000 Illumina Infinium probes and to 27,053 probes of the Affymetrix U133 Plus 2.0 platform. Visualization of multi-dimensional data was performed using the *SIGMA*^*2 *^software [29].

To determine the genetic events that caused (or could explain) gene expression status, we first identified a set of overexpressed and underexpressed genes for each cell line sample relative to MCF10A based on differential expression criteria mentioned above. Each cancer sample may have a different number of differentially expressed genes. Second, for each differentially expressed gene in each sample, we examined the copy number status, methylation status, and allelic status. A differential expression was considered "explained" when the observed expression change matched the expected change at the DNA level. If a gene was overexpressed, the causal copy number status would be a gain, DNA methylation status would be hypomethylation, or allelic status would be allelic imbalance. Conversely, if a gene was underexpressed, the causal copy number status would be a loss, DNA methylation status would be hypermethylation, or allelic status would be LOH. From this point forward, when a change in allele status with overexpression is discussed, it will be denoted as allelic imbalance (AI). Conversely, for underexpression, a change in allele status will be denoted as loss of heterozygosity (LOH). While changes in methylation or changes in gene dosage leading to differential expression are more commonly discussed, previous studies have shown that changes in allele status without change in copy number (copy neutral AI or LOH) can also lead to differential gene expression due to preferential allelic expression [30-32].

### Multiple concerted disruption (MCD) analysis

To determine what are likely key nodes in pathways and functions, we hypothesize that, in addition to being altered frequently (by one mechanism or multiple mechanisms), these genes also exhibit multiple concerted disruption (MCD) in a given sample. That is, a congruent change in gene copy number (gain or loss) accompanied by allelic imbalance and change in DNA methylation (hypomethylation or hypermethylation) resulting in a change in gene expression (over or underexpression). Moreover, the MCD events would be used as a similar screening approach to gene amplifications (multi-copy increases) or homozygous deletions whereby the expectation is that these events would occur at a lower frequency than disruptions through one mechanism alone and observation of these events would signify importance to the genes in question.

In this study, the MCD strategy can be broken down into four sequential steps. First, using a pre-defined frequency threshold, we identify a set of the most frequently differentially expressed genes. Second, we identify the most frequently differentially expressed genes from step 1 whose expression change is frequently associated with concerted change in at least one DNA dimension (either DNA copy number, DNA methylation or allelic status) within the same sample. Next, we further refine this subset of genes from step 2 by selecting those having concerted change in all dimensions in the same sample which we term as MCD. Finally, we introduce an additional level of stringency by requiring a minimum frequency of MCD in the given cohort. At the end of the process, we identify a small subset of genes which exhibit disruption through multiple mechanisms and show consequential change in gene expression.

### Simulated data analysis

Using the status of DNA alteration and expression for every gene in every sample, data within each sample were shuffled and randomized ten times to create ten simulated datasets. Each dataset was analyzed for overall disruption frequency and MCD and all results were then aggregated to determine the frequency distribution of different thresholds observed in the randomized data analysis.

### Pathway enrichment analysis

For pathway analysis, *Ingenuity Pathway Analysis *software (version 8.5) was used (Ingenuity Systems, CA, USA). Specifically, the core and comparison analyses were used, with focus on canonical signaling pathways. Briefly, for a given function or pathway, statistical significance of pathway enrichment is calculated using a right-tailed Fisher's exact test based on the number of genes annotated, number of genes represented in the input dataset, and the total number of genes being assessed in the experiment. A pathway was deemed significant if the p-value of enrichment was ≤ 0.05 (adjusted for multiple comparisons using a Benjamini-Hochberg correction).

### Survival and differential gene expression analysis in publicly available datasets

For survival analysis, Kaplan-Meier analysis was performed using the statistical toolbox in *Matlab *(Mathworks). For each gene, the expression data were sorted from lowest to highest expression across the sample set and survival times were compared between the top 1/3 and bottom 1/3 of the samples. Two publicly available gene expression microarray datasets with survival data were utilized for this analysis [4,33]. For the Sorlie *et al *dataset, individuals whose cause of death was not breast cancer were excluded from the analysis and missing data due to quality control issues were filled using the knn method in the "impute" package in *Bioconductor *[34]. Of the 23 genes selected by our MCD analysis (see Results), 17 were represented in either dataset. Survival distributions were compared using a log rank test and two-tailed p-values unadjusted for multiple comparisons were reported. Log-rank test code was obtained from Matlab File exchange http://www.mathworks.com/matlabcentral/fileexchange/22317-logrank.

Subsequently, these 17 genes were further evaluated for differential expression in publicly available expression datasets of clinical breast cancer samples using the *Oncomine *database [35].

## Results and Discussion

### Analysis of individual genomic dimensions

**Figure 1:**
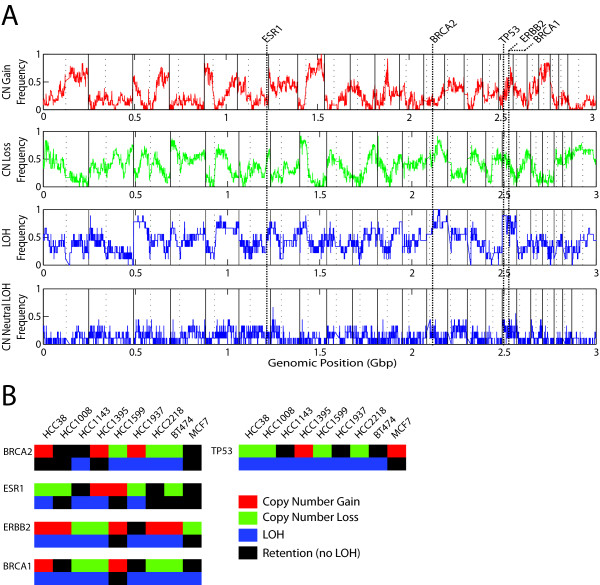
**Genomic profiles of breast cancer cell lines**. **(A) **Whole genome frequency analysis copy number gain (red), copy number loss (green), loss of heterozygosity/allelic imbalance (AI) (top blue) and copy number neutral LOH/AI (bottom blue). Vertical lines through all four graphs represent the genomic location of key breast cancer genes, using the hg18 build of the human genome map. **(B) **Illustration of copy number and LOH/AI status for *ESR1*, *BRCA1*, *BRCA2*, *ERBB2 *and *TP53 *in each of the samples. Each of these DNA events is evident in all of these genes.

When examining each genomic dimension alone, we see that many of the common features identified are consistent with the current knowledge of breast cancer genomes, for example, previously reported chromosomal regions of frequent copy number gain, segmental loss and loss of heterozygosity (LOH)/allelic imbalance (AI) (Figure 1A) [6,8,11,12,36]. While many regions of frequent LOH/AI do overlap with regions of copy number change, others are in regions of neutral copy number. Key genes implicated in breast cancer reside in these specific regions and are altered expectedly (Figure 1B).

### Multi-dimensional analysis (MDA) reveals a higher proportion of intra-sample deregulated gene expression can be explained when more dimensions are analyzed

**Figure 2:**
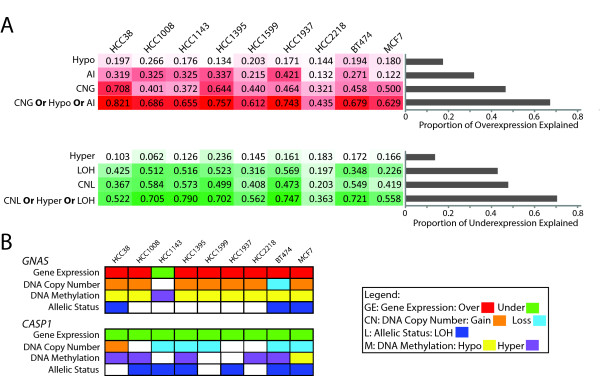
**Quantitative and qualitative benefits of integrative analyses**. **(A) **Heatmap and bar plot illustration of the additive benefit of multi-dimensional DNA analysis for the explanation of consequential differential gene expression. Within a sample, when sequentially adding a DNA dimension of analysis, an increasing percentage of observed differential gene expression can be explained. For each dimension or combination of dimensions, in the bar plot, the median value is used (grey bars). Heatmaps display the percentage of differential expression explained by DNA mechanisms, with values near to 100 either dark red (overexpression) or green (underexpression) and values closer to 0 in white. **(B) **Two specific genes *GNAS *and *CASP1 *are given as examples to show multiple and complementary mechanisms of gene disruption, illustrating the importance of multi-dimensional analysis (MDA).

The impact of integrative, multi-dimensional analysis on gene discovery is observed at two levels: (i) within an individual sample as well as (ii) across a set of samples. Within a given sample, we see that by sequentially examining more genomic dimensions at the DNA level, i.e. gene dosage, allelic status, and DNA methylation, we can explain a higher proportion of the differential gene expression changes observed. Interestingly, although this proportion may vary between samples, it always increases with every additional dimension examined (Figure 2A). For example, in HCC1395, a single genomic dimension alone can explain as much as 64.4% of overexpression but when using all three DNA based dimensions, whereby gene overexpression can be explained by disruption at the DNA level in at least one dimension, as much as 75.7% of aberrant overexpression can be explained. Similarly, in HCC1937, an increase from 56.9% to 74.7% explainable underexpression is observed when moving from one to three genomic dimensions respectively. Conversely, in HCC2218, we observe 44% and 36% of overexpression and underexpression respectively when using all three DNA dimensions. This suggests that the majority of differential expression in sample HCC2218 is most likely a result of complex gene-gene trans-regulation and consequently, highlights the individual differences between samples.

### MDA reveals genes are disrupted at higher frequencies when examining multiple dimensions as compared to any single dimension alone

When considering across a sample set, we see that analysis of multiple genomic dimensions leads to the discovery of more disrupted genes than what would be detected using a single dimension of analysis alone. For each identified gene, we gain insight in how multiple mechanisms are complementary in gene disruption (Figure 2B). For example, the tumor suppressor gene *caspase 1 *(*CASP1*) has been thought to be deactivated through DNA hypermethylation in multiple cancer types [37,38]. The gene is underexpressed in all nine cases examined in this study. In a subset of these cases, the observed underexpression can be attributed to copy number loss. Interestingly, in the remaining cases, DNA hypermethylation and copy neutral LOH are observed. Similarly, in another example, *GNAS *is differentially expressed in all nine cases, with a subset of cases showing concerted copy number change while the remaining cases reveal concerted change in DNA methylation. Notably, our conclusion is supported by recent studies of glioblastoma, that also showed higher than expected disruption frequencies of specific genes when multiple genomic dimensions were analyzed [39,40]. These examples illustrate how deregulated genes can be detected in more cases when multiple, but complementary, approaches are used.

Until very recently, multi-dimensional genomic analysis typically represented the parallel examination of gene dosage and gene expression. To demonstrate the power of examining multiple dimensions, we examine the frequency of gene expression deregulation explained by congruent alteration at the DNA level. Briefly, for each gene, a sample is determined to have a DNA explained gene expression change if any of the following criteria are met; gene overexpression should be accompanied with either (i) copy number gain, (ii) copy neutral allelic imbalance, or (iii) hypomethylation and gene underexpression should be accompanied with either (i) copy number loss, (ii) copy neutral LOH, or (iii) hypermethylation.

**Figure 3:**
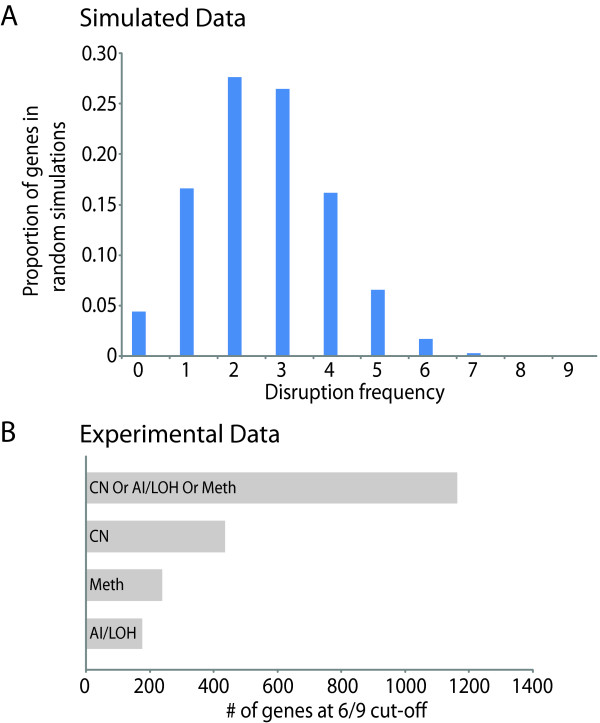
**Determination and application of a disruption frequency threshold**. **(A) **Results of the analyses of ten simulated datasets. Aggregating the results of the simulated analyses, the proportion of genes in random simulations at the observed frequency thresholds are shown. From these analysis, approximately 2% of the simulations were ≥ 6/9. **(B) **Using a frequency cut-off of 6/9, the number of genes disrupted at that frequency using a single or combination of DNA dimensions. With a single dimension alone, we can maximally identify 437 genes which are differentially expressed and exhibit a concerted change at the DNA level in a minimum of 6/9 samples. However, using all three dimensions, we find that 1162 genes are in fact differentially expressed and contain at least one concerted change in one of the DNA dimensions. This represents over a two-fold increase in the number of genes identified.

To determine an appropriate frequency of disruption threshold, ten random, simulated datasets were generated and a distribution plot was generated for all of the observed frequencies from 0/9 to 9/9 across all simulations (Figure 3A). The proportion of observed frequencies ≥ 5/9 was 0.086 but for ≥ 6/9, the proportion was 0.020. Thus, since the 6/9 threshold was the first threshold ≤ 0.05, 6/9 was used for further analysis. Using this threshold, we found that 437 differentially expressed genes have a corresponding change in gene dosage. Scaling this approach to examining the whole genome at multiple dimensions, we anticipate identifying more disrupted genes. When we added the remaining dimensions to account for differential expression, at the same frequency cut-off, we identified the mechanism of disruption for 1162 deregulated genes (Figure 3B, Additional File 4).

**Figure 4:**
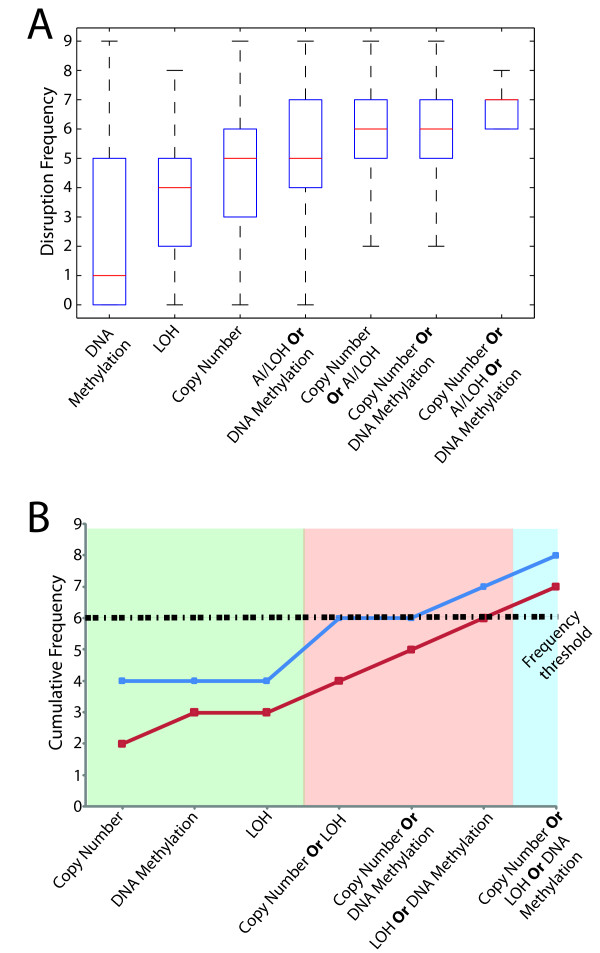
**Impact of multi-dimensional analysis on low frequency events**. **(A) **Box plot analysis of the frequency distribution of single and multi-dimensional analyses (MDA) of the 1162 genes differentially expressed with a concerted change in one of the DNA dimensions. The area in red represents the number of genes (of the 1162) that would be missed if only a single DNA dimension was examined, while the area in blue represents the genes that would be detected. Examining the median values for the three right-most boxes, we see that by even using the box with the highest median (copy number), we would not be able to detect about 50% of the 1162 genes. **(B) **Two specific examples highlighting the importance of multi-dimensional genomic analysis. Using single dimensional analyses (green shade) alone, *CD70 *(blue line graph) and *ENG *(red line graph) disruption occur at very low frequencies (44% and 33% respectively). However, when examining two (red shade) or three genomic dimensions (blue shade), the disruption of these genes occurs at very high frequencies, 88% and 77% respectively. Frequency threshold of 6/9 is denoted with a black dotted line.

The impact of multi-dimensional integrative analysis on cancer gene discovery is the enhanced detection of genes which are disrupted by multiple mechanisms but at lower frequencies for individual mechanisms. Collectively, the detection of gene dosage, allelic conversion and change in methylation status enable the identification of such genes as frequently disrupted. Using the list of 1162 genes, the distributions of alteration frequencies for each genomic dimension or combination of dimensions were assessed (Figure 4A). Examining the median frequencies in each box plot, there is a sequential increase in the median as more dimensions are examined. This point can be further validated using specific genes. For example, the *CD70 *and *ENG *genes are underexpressed in the majority of samples. Using copy number analysis alone, the observed frequency of disruption (loss and underexpression) is 44% and 22% respectively. If we then examine the methylation status, in the remaining cases not explained by DNA copy number, we observe an additional 33% of cases exhibiting hypermethylation and underexpression for *ENG *(red) and 22% for *CD70 *(blue). Finally, when we also examine allelic status, we observe an additional 22% of cases with copy neutral LOH and gene underexpression for *CD70 *and 11% for *ENG*. In total, by using all three dimensions, the cumulative frequency of disruption is 88% for *CD70 *and 77% for *ENG *(Figure 4B). This example demonstrates the utility of a multi-dimensional approach to elucidate events which would escape conventional single dimensional analysis.

### MDA identifies significantly enriched cancer related pathways

**Figure 5:**
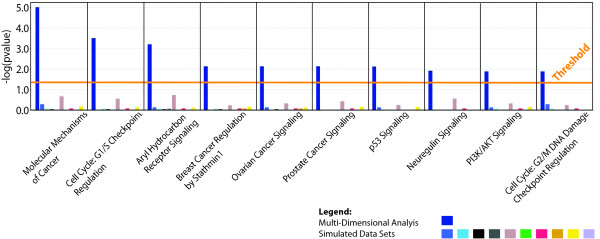
**Pathway analysis of the 1162 genes identified by multi-dimensional analysis**. *Ingenuity Pathway Analysis *of the 1162 genes identified by MDA as well as genes meeting the same frequency criteria (6/9) from the analysis of the ten simulated datasets. In total, using the list of 1162 MDA genes, 53 canonical signaling pathways were identified as significant after multiple testing correction using a Benjamini-Hochberg correction (Additional File 5). In contrast, using the same statistical criteria, nine of the 10 simulated datasets yielded no significant pathways with one of the datasets yielding one pathway. In this figure, ten of the most well known, cancer-related pathways are shown. The yellow threshold line represents a Benjamini-Hochberg corrected p-value of 0.05 with bars above that line deemed significant. The first blue bar represents the analysis of the actual dataset and the subsequent ten bars represent the analyses of the ten simulated datasets.

Using the set of 1162 genes identified by MDA (Additional File 4) and the similar lists of genes identified from each of the simulated datasets, pathway analyses were performed with *Ingenuity Pathway Analysis*. From the pathway analysis of MDA genes and focusing only on canonical signaling pathways, 53 pathways were significantly enriched for at a Benjamini-Hochberg corrected p-value of 0.05 (Additional File 5). In contrast, using the gene lists from the 10 simulated datasets, nine of the 10 pathway analyses yielded no significant pathways enriched for at the same p-value with one of the pathway analyses yielding one significant pathway. Similar results from *Gene Ontology *analysis were obtained using the publicly available *GATHER *database [41] (Additional File 6). Specific pathways involved in breast cancer, ovarian cancer, and prostate cancer were amongst the ones identified as most significant (Figure 5). Consequently, these results suggest that the genes identified using MDA have a high degree of biological relevance.

### MDA of the Neuregulin signaling pathway reveals a complex pattern of deregulation

**Figure 6:**
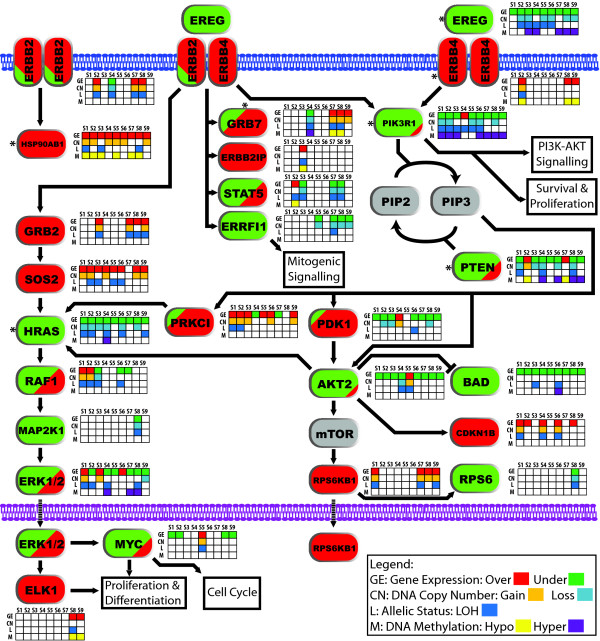
**Complex deregulation of the Neuregulin/ERBB2 signaling pathway**. Each gene is color-coded red and green to represent over and underexpression respectively. Genes colored both represent genes which are over and underexpressed in different samples. Beside each gene is the status for gene expression, copy number, LOH/AI and DNA methylation, with the alterations in each dimension colored as per the legend. DNA alterations are only shown when a change in gene expression is observed. It should be noted that LOH can be derived from multiple mechanisms. In this study, we do not distinguish between the which mechanisms. Likewise, methylation changes may affect one or both alleles. In this study, we do not distinguish the status of the alleles individually. Genes denoted with * have one sample exhibiting multiple concerted disruption (MCD). Samples are coded as follows: S1 = HCC38, S2 = HCC1008, S3 = HCC1143, S4 = HCC1395, S5 = HCC1599, S6 = HCC1937, S7 = HCC2218, S8 = BT474, and S9 = MCF7.

Among the 53 pathways which were statistically over-represented from our list of 1162 genes, one of the pathways identified is the neuregulin pathway. This pathway contains the well known breast cancer oncogene ERBB2 as well as other genes known to be affected in breast and other cancers [42-45]. Examining the components of this pathway, we observe that some are genes commonly altered while others are infrequently altered across our sample set by multiple patterns of genomic alteration, and some genes which behave oppositely in different samples (Figure 6).

**Figure 7:**
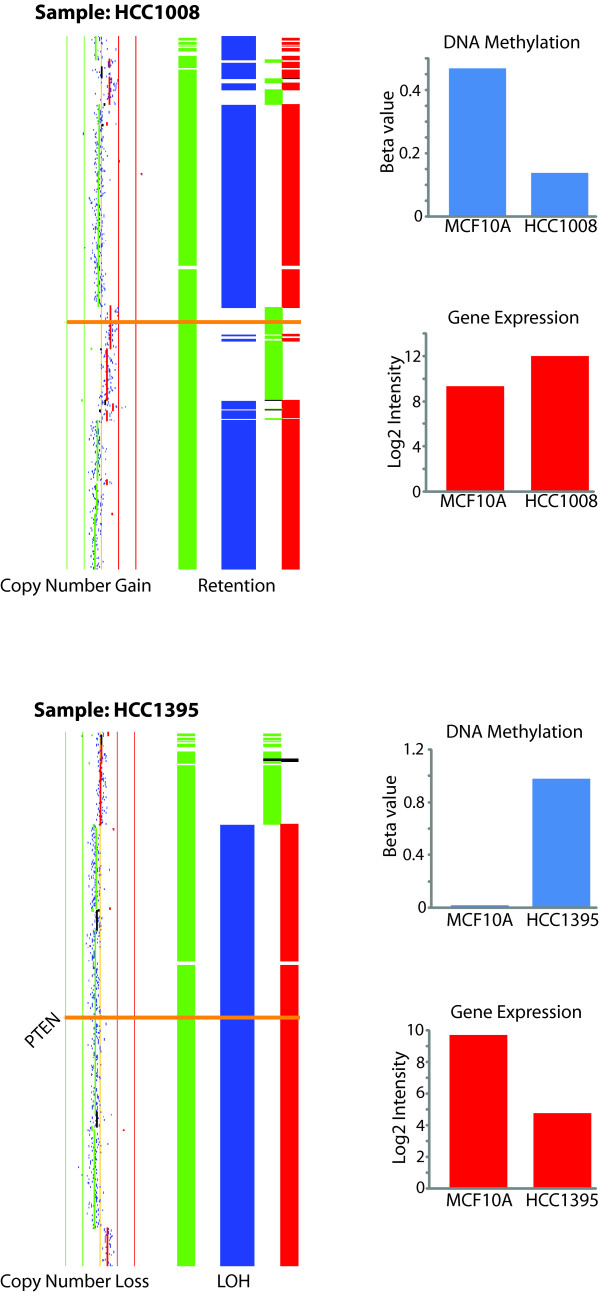
**Deregulation of *PTEN *occurs differently between samples**. In HCC1008 (top), *PTEN *is overexpressed with an associated gain in copy number and hypomethylation. Conversely, in HCC1395 (bottom), *PTEN *is underexpressed, with an associated loss in copy number, LOH, and DNA hypermethylation. This illustrates how each tumor may behave differently from another.

While genes such as *HRAS *(down), *BAD *(down), *HSP90AB1 *(up), *SOS2 *(up) and *RPS6KB1 *(up) generally exhibit consistent differential expression with concerted change at the DNA level across our sample set, genes such as *GRB7*, *PTEN*, and *MAP2K1 *exhibit both overexpression and underexpression, with concerted DNA change, in different samples. For example, if we examine PTEN, we observe copy number loss, LOH, DNA hypermethylation and consequent underexpression in HCC1395 while HCC1008 contains copy number gain, with DNA hypomethylation and consequent overexpression (Figure 7). The impact of such a difference on a downstream targets was recently shown in a breast cancer study where AKT and mTOR phosphorylation were higher in cases with low PTEN expression compared to those with high PTEN expression [46]. Using this pathway as an example, though average features across a sample set are important, those differences between samples in the same pathway may also play an important role and thus, may have a consequence on the biology of the tumor.

### Genes exhibiting multiple concerted disruption (MCD) - biological and clinical significance

We have demonstrated that we can identify more disrupted genes in a given sample when considering any mechanism of disruption. On the other hand, those genes which exhibit multiple concerted disruption (MCD) across all DNA dimensions -- i.e. overexpression of a gene due to increased gene dosage, which led to allelic imbalance, and DNA hypomethylation at the same locus relieving regulation -- may likely have strong biological significance. Likewise, underexpression due to reduced gene copy number, resulting in LOH, and complementary DNA hypermethylation, leading to gene silencing may also be significant. By employing multiple dimensions of interrogation, genes exhibiting MCD are captured.

**Figure 8:**
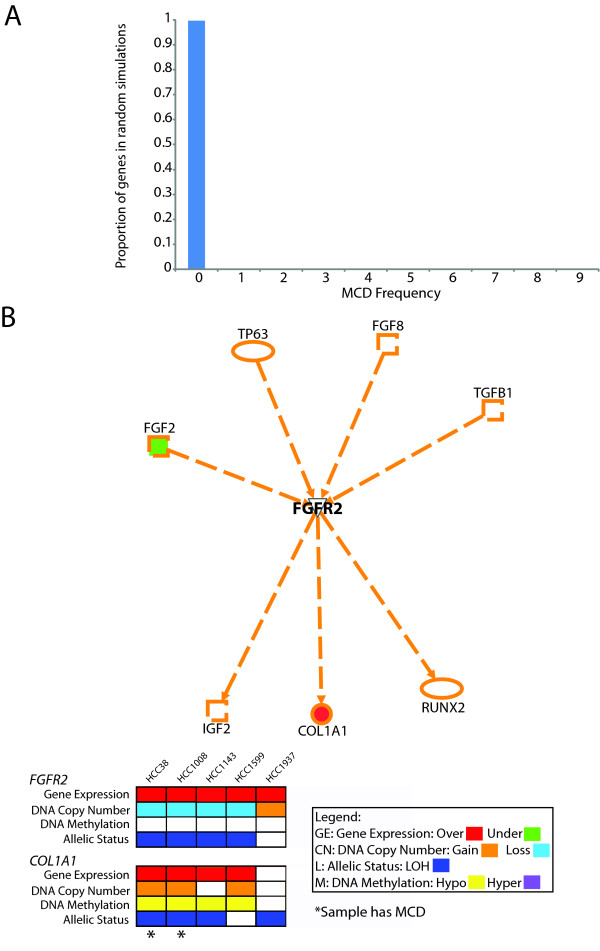
**Multiple concerted disruption (MCD) analysis and its application to triple negative breast cancer**. **(A) **Analysis of ten simulated datasets to determine the proportion of random simulations at each observed frequency of MCD. Notably, 99.7% of random simulations had a MCD frequency of 0/9, with the remaining 0.3% at 1/9. Moreover, no simulations showed a frequency ≥ 2/9. Thus, the observation of an MCD event suggests the event is likely non-random. **(B) **Using the knowledge database of *Ingenuity Pathway Analysis*, upstream and downstream components of FGFR2 were selected to assess their role in the subset of triple negative breast cancer (TNBC) cell lines. Only components which were shown to have a direct or indirect expression level relationship were selected. Of the seven components identified (four upstream and three downstream of *FGFR2*), one upstream and one downstream component were present in both the MDA list (Additional File 4) and MCD list (Additional File 7). Examining *FGFR2 *and *COL1A1*, while *FGFR2 *overexpression is not frequently associated with DNA level alteration, *COL1A1 *is frequently affected at DNA level. Moreover, in the five TNBC cell lines examined, four have DNA level alteration of *COL1A1 *and the remaining line has DNA level alteration of *FGFR2*.

To determine what frequency of MCD was deemed significant, we performed a similar analysis of the 10 simulated datasets from before and assessed the proportion of events at each frequency of MCD from 0/9 to 1/9 (Figure 8A). It was found that by random chance, a gene exhibiting MCD in 1/9 would occur 0.3% of the time. Thus, using this threshold of at least one MCD event, 974 genes were identified (Additional File 7). Interestingly, the overlap of the MDA list (1162 genes) with the MCD list (974 genes) yielded 375 genes.

The MCD strategy sequentially refines the roster of target genes with the intent of identifying critical genes for tumorigenesis (Additional File 8). Such genes which exhibit multiple mechanisms of deregulation, for example, may represent important nodes in pathways such as hub proteins [47], whereby disruption of the gene has an effect on multiple downstream targets or genes with biological and/or clinical relevance. Thus, although these genes may not be affected at a high frequency across the sample set, their disruption at multiple levels in individual samples would signify importance in tumorigenesis. As shown earlier, 375 genes identified by both MDA and MCD. If we further employed a criterion of frequent MCD, whereby this event occurs in 4/9 of cases (signifying high recurrence), we detect 23 genes (Additional File 8). Among the 23 genes identified are *TUSC3 *(8p22), *ELK3 *(12q23), and *CCNA1 *(13q12.3-q13).

*TUSC3 *resides at 8p22, a locus frequently deleted across multiple epithelial cancers [48-51]. ELK3 is an ETS domain transcription factor which, in mice, acts as a transcriptional inhibitor in the absence of RAS, but is a transcriptional activator in the presence of RAS [52]. Recently, *ELK3 *was shown to be underexpressed in a panel of breast cancer lines as well clinical breast tumor specimens [53]. *CCNA1 *was shown to be hypermethylated in multiple cancer types, including breast cancer [54].

To validate the relevance of the 23 MCD genes in clinical breast cancer samples, we evaluated gene expression levels associated with survival and examined multiple publicly available microarray datasets using the *Oncomine *database [35]. Of these 23 genes, 17 were represented in either the van de Vijver *et al *or Sorlie *et al *datasets. Interestingly, eight of these genes, demonstrated a statistically significant association with patient survival in at least one of the two independent datasets (Additional Files 9, 10) [4,33]. Moreover, when comparing the percentage of survival-associated genes (8/17, 47.1%) in the MCD gene list with what was expected without pre-selection (27.1%), the increased percentage was statistically significant based on the binomial test (p = 0.04131806). To further evaluate the clinical significance of these genes, we utilized the *Oncomine *database (Additional File 9). It should be noted the caveat of the *Oncomine *analysis is that it may not detect all low levels of differential expression. *TUSC3 *is shown as an example of one of the genes whose expression correlates with survival (Additional File 8, also see Methods). Notably, in ovarian cancer, *TUSC3*, in conjunction with *EFA6R*, also correlated with poor survival [55]. The observations that *TUSC3 *is altered frequently by multiple mechanisms at the DNA and RNA level and shows a strong association with patient survival, highlight the use of MCD in systematically identifying biologically, and potentially clinically, relevant genes.

### Association of genes exhibiting MCD and triple negative breast cancers (TNBC)

In this study, the majority of samples used (5/9) were of the triple negative subtype of breast cancer; a subtype which is estrogen receptor (ER) negative, progesterone receptor (PR) negative, and HER2 negative and represents between 10% and 20% of all diagnosed breast malignancies [56-59]. Genomic analyses of triple negative breast cancers (TNBCs) have been previously performed [60-63] and they revealed a heterogeneous and complex view of this breast cancer subtype. A recent study, however, had implicated fibroblast growth factor receptor 2 (FGFR2) as novel therapeutic target amplified in TNBCs [59]. Interestingly, from a meta-analysis of array CGH data, this gene was found to be amplified in 4% of TNBC cases [59]. Thus, we assessed the status of FGFR2 and its downstream targets in our multi-dimensional dataset.

While *FGFR2 *is not amplified in any of the five TNBC cell lines, all of the five cell lines showed overexpression of *FGFR2 *with one of the cell lines exhibiting a low level gain of a region encompassing *FGFR2 *(HCC1937). From this analysis, within the sample set of TNBC cell lines, though *FGFR2 *is overexpressed, it was not frequently associated with DNA level alterations.

However, examining downstream targets of FGFR2 revealed a striking finding. Using the knowledge database of *Ingenuity Pathway Analysis*, one of the downstream components affected at the expression level, which was also on both the MDA (Additional File 4) and MCD (Additional File 7) lists, was *COL1A1*. Remarkably, of the five TNBC cell lines, four exhibited DNA alteration associated overexpression of *COL1A1 *(two lines exhibited MCD at *COL1A1 *and two other lines have DNA copy number associated overexpression). The remaining line exhibited DNA copy number associated overexpression of *FGFR2 *(Figure 8B). Hence, every TNBC line was affected at either *FGFR2 *or *COL1A1*. Interestingly, *COL1A1 *has been shown to be both prognostic and predictive in multiple cancer types, including breast cancer [3,5,64,65].

## Conclusions

In conclusion, we have demonstrated that a multi-dimensional genomic approach is superior to analysis of one or two genomic dimensions alone. Each additional genomic dimension surveyed increases the amount of aberrant gene expression that can be explained within individual samples. As a by-product, when examining across a sample set, multi-dimensional genomic analysis can identify relevant genes that may be overlooked due to low frequencies of disruption by the individual mechanisms. The increased frequency of gene disruption detected, due to the consideration of multiple mechanisms of disruption, could potentially reduce the sample size of study cohort needed for gene discovery.

Secondly, while the increased detection of genes disrupted using multi-dimensional analysis is useful for achieving a more comprehensive identification of deregulated pathways and gene networks, it also presents a challenge in prioritizing which genes are likely key nodes or hubs in the affected pathways and networks. Hence, one way to prioritize is to identify genes with evidence of multiple concerted disruption. The Knudson two-hit hypothesis suggests that tumor suppressor genes require two allelic hits to disrupt gene function. Bi-allelic alteration, such as homozygous deletion, or concerted genetic and epigenetic changes, are well documented causal mechanisms of gene disruption. Likewise, hypomethylation and increased gene dosage are known mechanisms for gene overexpression. The bi-allelic disruption phenomenon (leading to loss or gain of function) provides a means to identify causative genes; hence, parallel analysis of the genome and epigenome in the same tumor is of great benefit. In this study, we have developed a stepwise gene selection strategy to identify multiple concerted disruptions using an integrative genomics approach.

In this study, three DNA dimensions, which have current affordable high throughput assays, were examined. However, we envision that new techniques for analysis of additional aspects such as histone modification states and gene mutation status will reveal mechanisms that would explain even more gene expression changes within individual samples. The identification of a number of key cancer-related genes and pathways using a relatively small sample size suggests that limitations in requiring large sample sizes for studies to identify relevant genes and pathways may be circumvented by our comprehensive approach. Consequently, this concept can be projected to current technologies such as high throughput sequencing where it may prove more prudent to perform this analysis in multiple dimensions in a smaller number of samples rather than in one dimension in many more samples at a comparable cost. Finally, observing the same gene in a given pathway being deregulated in a completely different manner between samples highlights one of the shortcomings of group-based analysis and highlights the eventual need to move to systems analysis of tumors as individual entities.

## Authors' contributions

RC designed the study, performed the analysis and wrote the manuscript. BPC contributed to data interpretation, study design and manuscript preparation. EAV provided technical assistance and contributed to manuscript preparation. WWL contributed to data interpretation and manuscript preparation. WLL is the principal investigator of this project. All authors have read and approved the final manuscript.

## Supplementary Material

Additional file 1 **Description of cell lines**. Background information about the cancer cell lines including mutation status and HER2/ER/PR statuses Click here for file

Additional file 2 **Sources of Data**. Listing of all the sources of data used in this paper. Public sources and newly generated data are indicated. Click here for file

Additional file 3 **Tab-delimited text file of the RMA normalized gene expression data**. Gene expression data file in a matrix format with all RMA normalized data points for each sample Click here for file

Additional file 4 **List of 1162 multi-dimensional analysis (MDA) genes altered in 6/9 samples by any DNA mechanisms with concerted change in gene expression**. A list of the 1162 genes identified by MDA. For each gene, the predominant status is listed. Description of the status is provided in the file. Click here for file

Additional file 5 **Canonical signaling pathways enriched using 1162 MDA genes**. Ingenuity Pathway Analysis of the 1162 genes Click here for file

Additional file 6 **Results of Gene Ontology analysis using *GATHER ***. Gene Ontology analysis of the 1162 genes using GATHER Click here for file

Additional file 7 **List of 974 multiple concerted disruption (MCD) analysis genes**. A list of the 974 genes exhibiting MCD in at least one sample. The predominant status is listed next to each gene. Click here for file

Additional file 8 **MCD strategy and Kaplan-Meier analysis of *TUSC3 ***. Overview of a strategy using MCD to identify relevant genes. Click here for file

Additional file 9 **Kaplan-Meier survival and *Oncomine *expression analyses of frequent MCD genes**. Summary of the Oncomine expression and K-M survival analysis of the 23 genes. Click here for file

Additional file 10 **Summary of Kaplan-Meier survival analysis**. Results of the K-M analyses using multiple datasets. Click here for file
