# Supplementary material for: An integrative multi-dimensional genetic and epigenetic strategy to identify aberrant genes and pathways in cancer
Source: BMC Syst Biol. 2010 May 17;4:67. doi: 10.1186/1752-0509-4-67 (PMC2880289; doi:10.1186/1752-0509-4-67)
Supplement: Additional file 9 — Kaplan-Meier survival and Oncomine expression analyses of frequent MCD genes. Summary of the Oncomine expression and K-M survival analysis of the 23 genes. [file 1752-0509-4-67-S9.DOC]

**Additional File 9. Kaplan-Meier and *Oncomine* expression analysis of frequent MCD genes)**

| *Symbol* | (+) | (-) | *Total* | *Survival Associated** | ***Status in Tumors (p-value)* |
| --- | --- | --- | --- | --- | --- |
| *SH3TC1* | 0 | 6 | 6 | No | - |
| *CCNA1* | 0 | 5 | 5 | Yes | - |
| *COL7A1* | 0 | 5 | 5 | No | - |
| *KCTD4* | 0 | 5 | 5 | N/A | Not tested |
| *LMCD1* | 5 | 0 | 5 | Yes | O3(6.3E-4), O5(3.1E-10) |
| *LYAR* | 0 | 5 | 5 | Yes | U5(3.8E-4), O3(2.6E-4) |
| *MTMR9* | 0 | 5 | 5 | No | U3(1.8E-7) |
| *SYT8* | 0 | 5 | 5 | N/A | Not tested |
| *TUSC3* | 0 | 5 | 5 | Yes | U5(7.4E-5) |
| *ASAM*  *B3GALNT1* | 0 | 4 | 4 | N/A | Not tested |
|  | 4 | 0 | 4 | No | O3(1.1E-7) |
| *COL17A1* | 0 | 4 | 4 | No | U1(6.8E-5), U3(1.4E-8) |
| *ELK3* | 0 | 4 | 4 | Yes | - |
| *FGFR1* | 0 | 4 | 4 | Yes | U1(2.6E-8),U3(4.2E-6),U4(1.3E-7) |
| *KRT17* | 0 | 4 | 4 | No | U1 (2.3E-11),U2 (1.1E-7), U3(3.9E-7) |
| *LCP1* | 0 | 4 | 4 | Yes | - |
| *OSBPL5* | 0 | 4 | 4 | N/A | Not tested |
| *PSD3* | 0 | 4 | 4 | Yes | - |
| *SFXN3* | 0 | 4 | 4 | N/A | Not tested |
| *SH3BGRL3* | 0 | 4 | 4 | No | O2(2.8E-4) |
| *SNRPN* | 0 | 4 | 4 | No | U3(5.4E-10), U5(2.7E-5) |
| *TNFRSF10D* | 0 | 4 | 4 | No | O5(5.2E-4), U3(9.7E-4) |
| *TNS4* | 0 | 4 | 4 | N/A | Not tested |

*Survival associated if gene expression was significant associated with survival in at least one of the two datasets tested (based on *p* < 0.05 using the log rank test).

**U=underexpressed between tumor and normal, O=overexpressed between tumor and normal in the particular dataset; The numbers 1-5 indicate the reports from which the data originated, 1= [1], 2= [2], 3=[3], 4=[4], 5=[5], 6=[6]; “-“ indicates gene was either not represented or not statistically differentially expressed based on group-wise analysis. (+) represents two-fold overexpression, copy number gain, hypomethylation and allelic imbalance; (-) represents two-fold underexpression, copy number loss, hypermethylation, and LOH in the same sample and the number of samples in our dataset which met this criteria.

**REFERENCES**

1. Sorlie T, Perou CM, Tibshirani R, Aas T, Geisler S, Johnsen H, Hastie T, Eisen MB, van de Rijn M, Jeffrey SS *et al*: **Gene expression patterns of breast carcinomas distinguish tumor subclasses with clinical implications**. *Proc Natl Acad Sci U S A* 2001, **98**(19):10869-10874.

2. Perou CM, Sorlie T, Eisen MB, van de Rijn M, Jeffrey SS, Rees CA, Pollack JR, Ross DT, Johnsen H, Akslen LA *et al*: **Molecular portraits of human breast tumours**. *Nature* 2000, **406**(6797):747-752.

3. Richardson AL, Wang ZC, De Nicolo A, Lu X, Brown M, Miron A, Liao X, Iglehart JD, Livingston DM, Ganesan S: **X chromosomal abnormalities in basal-like human breast cancer**. *Cancer Cell* 2006, **9**(2):121-132.

4. Radvanyi L, Singh-Sandhu D, Gallichan S, Lovitt C, Pedyczak A, Mallo G, Gish K, Kwok K, Hanna W, Zubovits J *et al*: **The gene associated with trichorhinophalangeal syndrome in humans is overexpressed in breast cancer**. *Proc Natl Acad Sci U S A* 2005, **102**(31):11005-11010.

5. Finak G, Bertos N, Pepin F, Sadekova S, Souleimanova M, Zhao H, Chen H, Omeroglu G, Meterissian S, Omeroglu A *et al*: **Stromal gene expression predicts clinical outcome in breast cancer**. *Nat Med* 2008, **14**(5):518-527.

6. Karnoub AE, Dash AB, Vo AP, Sullivan A, Brooks MW, Bell GW, Richardson AL, Polyak K, Tubo R, Weinberg RA: **Mesenchymal stem cells within tumour stroma promote breast cancer metastasis**. *Nature* 2007, **449**(7162):557-563.
